# Weight Stigma in Patients With Obesity and Its Clinical Correlates: A Perspective From an Indian Bariatric Clinic

**DOI:** 10.7759/cureus.26837

**Published:** 2022-07-14

**Authors:** Stephen A Jiwanmall, Dheeraj Kattula, Munaf B Nandyal, Sandhiya Parvathareddy, Richard Kirubakaran, Felix Jebasingh, Thomas V Paul, Nihal Thomas, Nitin Kapoor

**Affiliations:** 1 Psychiatry, Christian Medical College and Hospital, Vellore, IND; 2 Nutrition, Christian Medical College and Hospital, Vellore, IND; 3 Biostatistics, Centre for Biostatistic and Evidence Based Medicine, Vellore, IND; 4 Endocrinology, Diabetes and Metabolism, Christian Medical College and Hospital, Vellore, IND

**Keywords:** stress, psychiatric disorders, weight loss, obesity, stigma

## Abstract

Introduction

Obesity being a global epidemic, currently has several adverse health outcomes. Weight stigma is a significant barrier to delivering quality services and also impairs clinical progress. We intended to study the association of stigma with demographic and clinical variables in obese patients to identify the obstacles in treatment-seeking, so stigma could be adequately addressed to improve clinical outcomes.

Methods

This study was a retrospective chart review in a Bariatric clinic in a tertiary care hospital. The weight self-stigma questionnaire (WSSQ) was routinely used in the clinic. Demographic and clinical data were collected for 146 obese patients.

Results

Female patients (73%) had higher stigma scores. The mean total stigma score was 41.6(SD 3.83), the total self-devaluation score was 21.88(SD 2.10), total fear of enacted stigma was 21.26(SD 2.33). Multivariate analysis revealed an association between stigma with multiple dysfunctional eating patterns like bingeing, overeating, and grazing (Adjusted aOR 3.86, 95% CI- 1.66-8.96) and psychiatric diagnosis (adjusted aOR 3.00, CI- 1.25-7.17).

Conclusion

This study found an association between stigma and certain clinical variables that maintain and worsen obesity and comorbid psychiatric diagnoses. This highlights the importance of an assessment of mental health and stigma in general practice when dealing with patients with obesity. Treating the underlying psychiatric comorbidities and addressing unhealthy eating behaviors can help reduce self-stigma. Stigma is a barrier to treatment-seeking that needs to be addressed in the community.

## Introduction

Obesity has reached epidemic proportions globally and contributes to significant morbidity, mortality, and health care costs. In India, the prevalence rates vary from 11.8% to 31.3% [1]. 

The issue of stigma has substantial implications in the assessment and management of obesity. Weight stigma is defined as "discriminatory acts and ideologies targeted towards individuals because of their weight and size." Weight bias refers to the "negative ideologies associated with obesity” [2]. A lot of research work has been done on this over the past decade. The usual sources of these negative stereotypes are employers, peers, family members, and the media [3]. Individuals with obesity face significant social discrimination, bias, and prejudice even among health care providers [4]. Stigma among obese individuals causes significant psychological impairment and poor health determinants, ultimately leading to poor quality of life [3].

External devaluation often leads to negative self-perception and reduced self-esteem in individuals with obesity causing "internalized weight stigma [5]." Weight stigma, which in other studies has been termed weight bias internalization (WBI), is more prevalent in the obese than in the general population [6]. Although it is well established in scientific literature, there is a lack of information about the interaction between external and internal weight stigma. Despite its several implications, it has been inadequately assessed and addressed in routine clinical practice.

Weight stigma can be assessed through different methodologies like surveys, questionnaires with experimental manipulations, laboratory experiments, and field studies [7]. However, each of these methods has its advantages and disadvantages. Among the questionnaires, the weight self-stigma questionnaire (WSSQ) has been demonstrated with good reliability and validity and contains two distinct subscales-self-devaluation and fear of enacted stigma [8]. It can contribute to evaluating stigma reduction strategies in clinical settings.

We intended to study and assess weight stigma among obese individuals attending a bariatric clinic and its association with demographic and clinical variables. It was expected that the findings would bridge the gap in developing countries and help in managing the patient holistically by addressing the modifiable factors.

## Materials and methods

This retrospective chart review was conducted from the medical records of morbidly obese patients attending a multidisciplinary Bariatric clinic in a Medical College Hospital, a tertiary care referral center in South India. It caters to patients from all over India and neighboring countries. Only a small proportion of the patients are candidates for surgery. The treating team comprised endocrinologists, surgeons, dieticians, and psychiatrists.

Data collection

A team of three consultant psychiatrists assessed all patients attending the bariatric clinic for their first visit, and diagnoses were made using the International Classification of Diseases 10 criteria. Patients with obesity who were either referred to the clinic or registered themselves. A standardized proforma, which is adapted from the guidelines issued by the American Society for Bariatric Surgery was used routinely for assessment [9]. Information was recorded based on patient's self report. The information about dietary pattern, exercise, presence of adequate social support were based on patient's perception and not using any standardized scales for each of these, Additionally, WSSQ (Weight self-stigma questionnaire) was used to assess Weight stigma [8]. It is a 12-item self-report measure explicitly designed to determine weight self-stigma in overweight and obese individuals. It comprises of two subscales: self-devaluation (negative thoughts and emotions about being overweight) and fear of enacted stigma (involves esethe perception of being discriminated, as well as the identification of a stigmatized group). Items 1 to 6 comprise the self-devaluation subscale and items 7 to 12 are the fear of enacted stigma subscale. All items are rated on a 5-point Likert scale (1 = strongly disagree to 5 = strongly agree), with higher scores reflecting the presence of more weight self-stigma. The scale has high internal consistency with a Cronbach's α of 0.878, with subscale α's of 0.869 for the fear of enacted and 0.812 for self-devaluation subscales. Since the patients knew English, the questionnaire was filled by themselves. This information is stored under the psychiatric assessment section of the clinic records. 

The data collected from the medical records of the clinic from January 1, 2018, to December 31, 2018 were used for this study.

Statistical analysis

 For continuous data, the descriptive statistics Mean, SD, and for non-normally distributed interval data and ordinal data, median (interquartile range IQR) was presented. The number of patients and percentages were given for categorical data. The Chi-square test was used to find out the association between two categorical variables. Student t-test was used to find out the difference between the two groups. All tests were two-sided at the α=0.05 level of significance. Cut-off values for median total, high, and low stigma scores were based on the mean scores identified in a previous study in a treatment-seeking population (Total WSSQ mean 35.98(sd=8.69), WSSQ (self) mean 19.32(sd=4.38), WSSQ(enacted) mean 16.66(sd=5.61)[8]. All analysis was done using SPSS software Version 21.0 (Armonk, NY: IBM Corp).

Ethics

Approval from the Institutional review board was obtained to conduct this study (IRB min. 12652, dated 26/2/2020). The consent was waived because of study design being a retrospective chart review.

## Results

There were 184 patients with obesity who were seen during the study period. Some patients could not read English and a few had missed psychiatric evaluation. Records of 146 patients were complete and were included in the study. High stigma scores were seen more in females (73%). The mean total stigma score was 41.60(SD-3.83), the total self-devaluation score was 21.88(SD-2.10), total fear of enacted stigma was 21.26(SD-2.33). Baseline demographic characteristics, as described in Table 1, show no statistical significance with the stigma scores. 

**Table 1:** Demographic Characteristics n=number of subject; SD=standard deviation; * chi square test p value

		Low stigma (n=73)		High stigma (n=73)		p-value Chi-square/ T test
		mean / number	SD/percentage	mean / number	SD /percentage	
Age		41	13	38	12	0.194
Sex						
Male		25	34%	20	27%	0.370*
Female		48	66%	53	73%	
Height		161.5	9.6	160.3	8.5	0.429
Weight		100.0	18.3	105.2	18.7	0.093
BMI		39.0	7.2	46.4	48.8	0.204

Table 2 shows the stigma scores.

**Table 2:** Mean score levels in low and high stigma n=number of subject; SD=standard deviation; MD= mean difference; CI = confidence interval; LL = lower limit; UL = upper limit; ** p < .01

	Cut off value (Median score)	Low stigma			High stigma			MD	95% CI		p value
		n	Mean	SD	n	Mean	SD		LL	UL	
total stigma score	34	73	26.70	3.99	73	41.60	3.83	-14.90	-16.18	-13.62	0.001**
total self stigma -score	18	84	14.47	2.55	62	21.88	2.10	-7.41	-8.19	-6.62	0.001**
total enacted stigma score	16	79	12.50	2.08	67	21.26	2.33	-8.76	-9.48	-8.03	0.001**

Table 3 illustrates the different clinical characteristics assessed and their association with total stigma scores, self-devaluation stigma scores, and fear of enacted stigma scores. Patients with high total stigma scores had multiple dysfunctional eating patterns (n=39, 59%) and a statistically significant (p=0.009) association, despite 48 patients reporting no abnormal eating pattern. Presence of a psychiatric diagnosis (n=24,33%) was significantly associated with high stigma scores(p=0.021). The self-devaluation stigma scores were associated with multiple dysfunctional eating patterns (n=31, 50%) (p=0.045). Suicidal behaviors (n=6, 10%), although they were reported in a few patients but were significantly associated with high self-devaluation stigma scores (p= 0.018). The clinical variables which were significantly associated with high fear of enacted stigma scores were dysfunctional eating patterns (p=0.005), presence of psychiatric diagnosis (p=0.035), Suicidal behaviors (p=0.030), and psychosocial stressors (p=0.013).

**Table 3:** Clinical characteristics associated with Total Weight-stigma, Self-Devaluation subscale, and fear of enacted stigma subscale CI = confidence interval * p < .05

variables	Total weight stigma			Total self-stigma [cut off of 18]			Total enacted stigma [cut off of 16]		
	Low stigma	High stigma	Chi-square test	Low stigma	High stigma	Chi-square test	Low stigma	High stigma	Chi-square test
	(n=73)	(n=73)	p value	(n=83)	(n=63)	p value	(n=79)	(n=67)	p value
	n(%)	n(%)		n(%)	n(%)		n(%)	n(%)	
Dietary Patterns									
binge eating	2(3%)	3(4%)	0.009*	3(4%)	2(3%)	0.045*	3(4%)	2(3%)	0.005*
overeating	14(19%)	6(8%)		16(19%)	4(6%)		15(19%)	5(7%)	
grazing	4(5%)	8(11%)		4(5%)	8(13%)		5(6%)	7(10%)	
none	31(42%)	17(23%)		31(37%)	17(27%)		33(42%)	15(22%)	
multiple	22(30%)	39(53%)		30(36%)	31(50%)		23(29%)	38(57%)	
Exercise	19(26%)	21(29%)	0.711	23(27%)	17(27%)	0.996	22(28%)	18(27%)	0.894
Substance use	8(11%)	9(12%)	0.796	8(10%)	9(15%)	0.353	9(11%)	8(12%)	0.918
Risk behavior									
impulse control problems	4(5%)	9(12%)	0.146	6(7%)	7(11%)	0.384	4(5%)	9(13%)	0.077
none	69(95%)	64(88%)		78(93%)	55(89%)		75(95%)	58(87%)	
Cognitive deficits	2(3%)	0(0%)	0.154	2(2%)	0(0%)	0.221	2(3%)	0(0%)	0.19
Coping skills									
Healthy	59(81%)	53(75%)	0.24	66(79%)	46(74%)	0.536	65(82%)	47(70%)	0.084
Unhealthy	14(19%)	20(27%)		18(21%)	16(26%)		14(18%)	20(30%)	
Psychiatric diagnosis (Any including Dysthymia, Depressive Disorder, Psychotic disorder, Anxiety disorder)	12(16%)	24(33%)	0.021*	16(19%)	20(32%)	0.067**	14(18%)	22(33%)	0.035*
Suicidal behaviour	1(1%)	6(8%)	0.053**	1(1%)	6(10%)	0.018*	1(1%)	6(9%)	0.030*
Physical / sexual abuse	0(%)	1(1%)	0.316	0(0%)	1(2%)	0.243	0(0%)	1(1%)	0.276
Traumatic life event	13(18%)	18(25%)	0.312	18(21%)	13(21%)	0.946	12(15%)	19(28%)	0.053
Stressors	33(45%)	40(55%)	0.247	39(46%)	34(55%)	0.315	32(41%)	41(61%)	0.013*
Presence of social support	69(95%)	67(92%)	0.512	78(93%)	58(94%)	0.87	74(94%)	62(93%)	0.787
Motivation to lose Weight	68(93%)	65(89%)	0.383	76(90%)	57(92%)	0.76	72(91%)	61(91%)	0.984
Expectations regarding treatment									
realistic	63(86%)	55(75%)	0.093**	71(85%)	47(76%)	0.186	67(85%)	51(76%)	0.184
unrealistic	10(14%)	18(25%)		13(15%)	15(24%)		12(15%)	16(24%)	

Multivariate logistic regression analysis is shown in Table 4 after adjusting for the variable that was significantly associated with the higher scores in univariate analysis. Multiple dysfunctional eating patterns (aOR: 3.866; 95% CI, 1.668-8.96; P =0.002), presence of psychiatric diagnosis (aOR: 3.000; 95% CI, 1.6254-7.178; P =0.014) are significantly associated with high stigma. However, for high self-devaluation, stigma scores are related to dietary grazing type (aOR: 4.336; 95% CI, 1.081-17.392; P = 0.038). Additionally, the Multiple dysfunctional eating patterns (aOR: 4.550; 95% CI, 1.879-11.018; P =0.001) variable was significantly associated with fear of enacted stigma.

**Table 4:** Multivariate analysis: Total Stigma, Self-stigma, and Enacted stigma CI = confidence interval * p < .05

	Total stigma				Total self stigma				total enacted stigma			
	crude odds ratio	p value	Adjusted odds ratio (95% CI)	p value	crude odds ratio	p value	Adjusted odds ratio (95% CI)	p value	crude odds ratio	p value	Adjusted odds ratio(95% CI)	p value
Dietary Habit												
binge eating	2.74	0.30	3.51(0.51-24.31)	0.20	1.22	0.84	1.47(0.21-10.02)	0.69	1.47	0.69	2.218(0.30-16.63)	0.439
overeating	0.78	0.67	0.84(0.26-2.7)	0.76	0.46	0.22	0.49(0.13-1.76)	0.28	0.73	0.61	0.836(0.24-2.91)	0.778
grazing	3.65	0.06	4.63(1.14-18.78)	0.032*	3.65	0.06	4.34(1.08-17.39)	0.038*	3.08	0.09	4.031(0.99-16.34)	0.051
multiple	3.23	0.00	3.87(1.67-8.96)	0.002*	1.88	0.11	2.11(0.93-4.74)	0.07	3.64	0.00	4.55(1.87-11.01)	0.001*
Psych diagnosis	2.49	0.02	3(1.25-7.17)	0.014*	2.02	0.07	2.21(0.96-5.06)	0.06	2.27	0.04	1.813(0.66-4.94)	0.246
Suicidal behaviour	6.49	0.09	5.98(0.59-59.77)	0.13	8.89	0.05	7.18(0.77-66.71)	0.08	0.13	0.06	4.864(0.49-47.53)	0.174

## Discussion

This cross-sectional study was done primarily to assess and measure the stigma of obese patients in a multidisciplinary clinic. Secondly, we wanted to find the essential clinical variables associated with high stigma scores. The strength of our study is that we used WSSQ, which has good psychometric properties, to assess weight stigma [10]. It also has been found to have good reliability (Cronbach's α of 0.878, with subscale α's of 0.869 and 0.812 for fear of enacted and self‐devaluation subscales, respectively) and validity among different clinical populations [8,9,11]. There has not been a study to the best of our knowledge in Indian settings exploring the stigma of obese patients, especially in a multidisciplinary clinic. Hence, it is the first of its kind.

Although in our study, none of the sociodemographic variables were significantly related to high stigma other studies in the western community, about 20%, report high weight stigma in the general population. Sociodemographic variables that were associated with high stigma were a Caucasian race, low education, low income, and higher BMI [6].

The Indian study among obese women in North India estimated the prevalence to be 18.3% to 32.5% [12]. Among obese adults in the USA, it is estimated to be found in approximately 50% [6].

Stigma and weight bias among obese are often rooted in the misconception that body weight can be easily controlled by altering dietary patterns and physical activity alone [13]. There is a complex interplay of factors in the etiology such as genetics, epigenetics, environment, societal and medication-related; therefore, modifying dietary and physical activity patterns may not suffice to bring about long-lasting changes in weight [14].

Any patient-centric model for treatment should factor in motivation, opportunity, and capability [15]. Several studies have shown that high self-perceived stigma is associated with decreased motivation to diet and unhealthy eating behaviors [16]. It also translates to an inability to lose weight and more weight gain in the long term [17,18]. Our findings are consistent with other studies that categorically depict the association between some unhealthy eating patterns and high weight stigma [19-21].

A review of studies has established an association between psychological distress and weight stigma [22]. Another report found a significant and bidirectional association between obesity and depression, but moderate evidence for anxiety disorders and none for the other psychiatric disorders [23]. The etiology of depression in obesity is poorly understood, but some proposed mechanisms include body concerns and social ostracization [24,25]. In an Indian study, a third of obese patients had psychiatric disorders, and among them, depressive disorders constituted half of the population [26]. In our study, mental disorders consistently had an association with self-devaluation and fear of enacted stigma. It was found significant in the multivariate analyses as well. Another interesting finding was the significant association between suicidal behaviors and high stigma scores. It is due to the higher number of depressive disorders associated with the obese population and a higher degree of self-devaluation [26]. Treating the psychiatric comorbidity and addressing stigma would fit neatly into the SECURE model of obesity management encompassing Severity assessment, Etiological evaluation, Comorbidity workup, Urging change in life style, Role of medications and surgery and Expected goal setting [27].

Finally, stress, a well-known factor in the implication of Obesity, has several causal mechanisms [28]. It includes several biological, physiological, and psychological processes. One proposed model, called the cyclic-obesity/weight-stigma-based model(COBWEBS), considers weight-stigma and weight gain in a "vicious cycle” [18]. It occurs through dysfunctional eating behaviors and increased cortisol secretion, in which stress plays a significant part. In our study, stress seems to be significantly associated with fear of enacted stigma and not with self-devaluation.

Overall recent research has shown that weight stigma has adverse health consequences, and impairs progress in weight management interventions [29]. Despite the overarching ill effects of stigma, shockingly, some believe that stigma is beneficial in motivating Obese people to lose weight [30]. Most of the Obese are seen by community doctors and nurses, and if stigma is identified and addressed at the community level, it facilitates treatment in secondary and tertiary care.

The major limitation of our study was the cross-sectional design of the study with which we cannot determine the cause for stigma, given that there is a significant association with a psychiatric diagnosis, which can affect the perception of stigma.

## Conclusions

In a study of obese patients attending a bariatric clinic in south India, most patients had low stigma scores. High stigma scores were significantly associated with specific modifiable clinical variables. These include unhealthy eating behaviors, the presence of psychiatric diagnosis, suicidal behaviors, and psychosocial stressors.

Firstly, stigma being an underrecognized and undealt factor in Obesity needs the urgent attention of public health practitioners. It will enable them to identify one of the most significant barriers to treatment seeking. Appropriate action at the community level through education and screening will make it convenient for Obese patients to explore different therapeutic options available. Secondly, it is essential to address stigma even in specialist settings in obese patients for referring and initiating appropriate psychosocial and psychiatric interventions. These may not only help in the reduction of stigma but also improve long-term clinical weight loss.

## References

[1] Ahirwar R, Mondal PR (2019). Prevalence of obesity in India: a systematic review. Diabetes Metab Syndr.

[2] Weight Stigma (2021). Weight Stigma. https://www.worldobesity.org/what-we-do/our-policy-priorities/weight-stigma#:~:text=Weight%20stigma%20refers%20to%20the,negative%20ideologies%20associated%20with%20obesity.

[3] Puhl RM, Heuer CA (2010). Obesity stigma: important considerations for public health. Am J Public Health.

[4] (2020). http://www.who.int/entity/nutrition/publications/obesity/WHO_TRS_894/en/index.html..

[5] Ratcliffe D, Ellison N (2015). Obesity and internalized weight stigma: a formulation model for an emerging psychological problem. Behav Cogn Psychother.

[6] Puhl RM, Himmelstein MS, Quinn DM (2018). Internalizing weight stigma: prevalence and sociodemographic considerations in US adults. Obesity (Silver Spring).

[7] Ruggs EN, King EB, Hebl M, Fitzsimmons M (2010). Assessment of weight stigma. Obes Facts.

[8] Lillis J, Luoma JB, Levin ME, Hayes SC (2010). Measuring weight self-stigma: the weight self-stigma questionnaire. Obesity (Silver Spring).

[9] Sogg S, Lauretti J, West-Smith L (2016). Recommendations for the presurgical psychosocial evaluation of bariatric surgery patients. Surg Obes Relat Dis.

[10] Maïano C, Aimé A, Lepage G, Morin AJ (2019). Psychometric properties of the Weight Self-Stigma Questionnaire (WSSQ) among a sample of overweight/obese French-speaking adolescents. Eat Weight Disord.

[11] Erdogan Z, Kurcer MK, Kurtuncu M, Catalcam S (2018). Validity and reliability of the Turkish version of the weight Self-stigma questionnaire. JPMA J Pak Med Assoc.

[12] Agrawal P, Gupta K, Mishra V, Agrawal S (2015). The psychosocial factors related to obesity: a study among overweight, obese, and morbidly obese women in India. Women Health.

[13] (2020). Ending weight bias and the stigma of obesity. Nat Rev Endocrinol.

[14] Blüher M (2019). Obesity: global epidemiology and pathogenesis. Nat Rev Endocrinol.

[15] Kalra S, Arora S, Kapoor N (2021). The motivation-opportunity-capability model of behavioural therapy - the vital component of effective patient centric obesity management. JPMA J Pak Med Assoc.

[16] Brewis A, SturtzSreetharan C, Wutich A (2018). Obesity stigma as a globalizing health challenge. Global Health.

[17] Vartanian LR, Smyth JM (2013). Primum non nocere: obesity stigma and public health. J Bioeth Inq.

[18] Tomiyama AJ (2014). Weight stigma is stressful. A review of evidence for the Cyclic Obesity/Weight-Based Stigma model. Appetite.

[19] Palmeira L, Pinto-Gouveia J, Cunha M, Carvalho S (2017). Finding the link between internalized weight-stigma and binge eating behaviors in Portuguese adult women with overweight and obesity: The mediator role of self-criticism and self-reassurance. Eat Behav.

[20] Webb JB, Hardin AS (2016). An integrative affect regulation process model of internalized weight bias and intuitive eating in college women. Appetite.

[21] Palmeira L, Cunha M, Pinto-Gouveia J (2018). The weight of weight self-stigma in unhealthy eating behaviours: the mediator role of weight-related experiential avoidance. Eat Weight Disord.

[22] Alimoradi Z, Golboni F, Griffiths MD, Broström A, Lin CY, Pakpour AH (2020). Weight-related stigma and psychological distress: A systematic review and meta-analysis. Clin Nutr.

[23] Rajan TM, Menon V (2017). Psychiatric disorders and obesity: a review of association studies. J Postgrad Med.

[24] Mooney SJ, El-Sayed AM (2016). Stigma and the etiology of depression among the obese: an agent-based exploration. Soc Sci Med.

[25] Brewis AA, Han SY, SturtzSreetharan CL (2017). Weight, gender, and depressive symptoms in South Korea. Am J Hum Biol.

[26] Jiwanmall SA, Kattula D, Nandyal MB (2018). Psychiatric burden in the morbidly obese in multidisciplinary bariatric clinic in South India. Indian J Psychol Med.

[27] Kapoor N, Kalra S, Kota S, Das S, Jiwanmall S, Sahay R (2020). The SECURE model: a comprehensive approach for obesity management. JPMA J Pak Med Assoc.

[28] Tomiyama AJ (2019). Stress and obesity. Annu Rev Psychol.

[29] Puhl R, Suh Y (2015). Health consequences of weight stigma: implications for obesity prevention and treatment. Curr Obes Rep.

[30] Verma M, Das M, Sharma P, Kapoor N, Kalra S (2021). Epidemiology of overweight and obesity in Indian adults - A secondary data analysis of the National Family Health Surveys. Diabetes Metab Syndr.

